# *KRAS* and *MAPK1* Gene Amplification in Type II Ovarian Carcinomas

**DOI:** 10.3390/ijms140713748

**Published:** 2013-07-02

**Authors:** Mohammed Tanjimur Rahman, Kentaro Nakayama, Munmun Rahman, Hiroshi Katagiri, Atsuko Katagiri, Tomoka Ishibashi, Masako Ishikawa, Emi Sato, Kouji Iida, Naomi Nakayama, Noriyuki Ishikawa, Kohji Miyazaki

**Affiliations:** 1 Departments of Obstetrics and Gynecology, Shimane University School of Medicine, Enyacho 89-1, Izumo, Shimane 6938501, Japan; E-Mails: tanjim_dmc@yahoo.com (M.T.R.); munmun_dmc@yahoo.com (M.R.); hkata@med.shimane-u.ac.jp (H.K.); atsukata@med.shimane-u.ac.jp (A.K.); tomo0314@med.shimane-u.ac.jp (T.I.); m-ishi@med.shimane-u.ac.jp (M.I.); prettynanaone@gmail.com (E.S.); iida@med.shimane-u.ac.jp (K.I.); miyazaki@med.shimane-u.ac.jp (K.M.); 2 Department of Biocehmistory, Shimane University School of Medicine, Izumo, Shimane 6938501, Japan; E-Mail: nn0411@med.shimane-u.ac.jp; 3 Department of Organ Pathology, Shimane University School of Medicine, Izumo, Shimane 6938501, Japan; E-Mail: kanatomo@med.shimane-u.ac.jp

**Keywords:** type II ovarian carcinoma, *KRAS*, *MAPK1*, gene amplification, survival, MEK inhibitor

## Abstract

In this study, we examined the clinical significance of *KRAS* and *MAPK1* amplification and assessed whether these amplified genes were potential therapeutic targets in type II ovarian carcinoma. Using fluorescence *in situ* hybridization, immunohistochemistry, and retrospectively collected clinical data, *KRAS* and *MAPK1* amplifications were identified in 9 (13.2%) and 5 (7.4%) of 68 type II ovarian carcinoma tissue samples, respectively. Interestingly, co-amplification of *KRAS* and *MAPK1* seemed to be absent in the type II ovarian carcinomas tested, except one case. Active phospho-ERK1/2 was identified in 26 (38.2%) out of 68 type II ovarian carcinomas and did not correlate with *KRAS* or *MAPK1* amplification. There was no significant relationship between *KRAS* amplification and overall or progression-free survival in patients with type II ovarian carcinoma. However, patients with *MAPK1* amplification had significantly poorer progression-free survival than patients without *MAPK1* amplification. Moreover, type II ovarian carcinoma cells with concomitant *KRAS* amplification and mutation exhibited dramatic growth reduction following treatment with the MEK inhibitor PD0325901. These findings indicate that *KRAS*/*MAPK1* amplification is critical for the growth of a subset of type II ovarian carcinomas. Additionally, RAS/RAF/MEK/ERK pathway-targeted therapy may benefit selected patients with type II ovarian carcinoma harboring *KRAS*/*MAPK1* amplifications.

## 1. Introduction

Ovarian carcinoma is the most lethal gynecological malignancy in American [1] and Japanese women. Among ovarian carcinomas, high-grade serous ovarian carcinoma mostly presents at an advanced stage and has a low overall survival rate. Standard treatment involves aggressive cytoreductive surgery followed by platinum- and taxane-based chemotherapy [2,3]. Although initial response rates to platinum-based chemotherapy exceed 75%, most tumors will eventually recur and become refractory to treatment, with a median survival of less than five years [2,4]. Although there are well-established surgical and chemotherapeutic treatments for ovarian cancer, there is significant opportunity to develop drugs targeting specific molecular pathways and to reduce rates of metastasis or relapse. Drugs of this type would be particularly useful for recurrent disease that shows chemoresistance. Thus, there is an initial, preclinical need to improve our understanding of the molecular pathways underlying ovarian carcinogenesis.

Recent morphological, immunohistochemical, and molecular genetic studies have led to the development of a new paradigm for the pathogenesis and origin of epithelial ovarian cancer based on a dualistic model of carcinogenesis that divides epithelial ovarian cancer into two broad categories, designated as types I and II. Type I tumors include low-grade serous, low-grade endometrioid, clear cell, and mucinous carcinomas; these tumors develop in a stepwise fashion from well-established precursor lesions, such as borderline tumors and endometriosis. Type I tumors are relatively genetically stable and typically display a variety of somatic mutations in *KRAS*, *BRAF*, *PTEN*, *PIK3CA*, *CTNNB1*, and *ARID1A*, but very rarely *P53* [5]. Type II tumors comprise high-grade serous, high-grade endometrioid, malignant mixed mesodermal tumors (carcinosarcomas), and undifferentiated carcinomas. They are aggressive, present at advanced stages, and have a very high frequency of *TP53* mutations, but rarely harbor the mutations detected in type I tumors [5].

Recent genome-wide analysis by TCGA, single nucleotide polymorphism arrays identified *KRAS* and *MAPK1* as two of the most frequently amplified genes in high-grade serous ovarian carcinomas with the prototypic type in type II ovarian carcinomas [6]. Gene amplification represents one of the molecular genetic hallmarks of human cancer. Elucidating the molecular mechanisms of how amplified genes initiate and maintain malignant phenotypes and propel tumor progression is fundamental to understanding the molecular etiology of human cancer and its therapeutic implications. Gene amplification is an important mechanism that allows cancer cells to increase expression of driver genes, such as oncogenes, that are involved in growth regulation and genes responsible for drug resistance. Therefore, detection of gene amplification in tumors may be of diagnostic, prognostic, and/or therapeutic relevance for patient management.

Based on the above findings, we hypothesized that *KRAS* or *MAPK1* amplification may play an important role in type II ovarian carcinoma progression and that amplification may correlate more strongly with clinical parameters. Furthermore, type II ovarian carcinomas with *KRAS* or *MAPK1* amplification may be sensitive to a potential targeted therapeutic agent. To test this hypothesis, we undertook the current study to assess gene copy numbers of *KRAS* or *MAPK1* and to evaluate any prognostic significance in patients with type II ovarian carcinoma. In addition, we compared phenotypes in cultured type II ovarian carcinoma cell lines with various *KRAS* or *MAPK1* copy numbers after treatment using a selective MEK inhibitor.

## 2. Results

### 2.1. Identification of *KRAS* or *MAPK1* Amplification and Correlation with p-ERK Immunostaining

**Figure 1:**
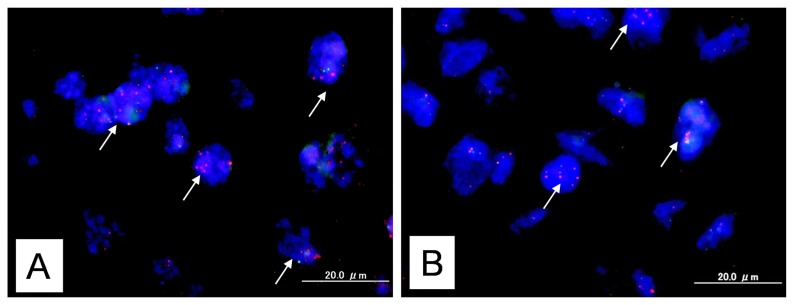

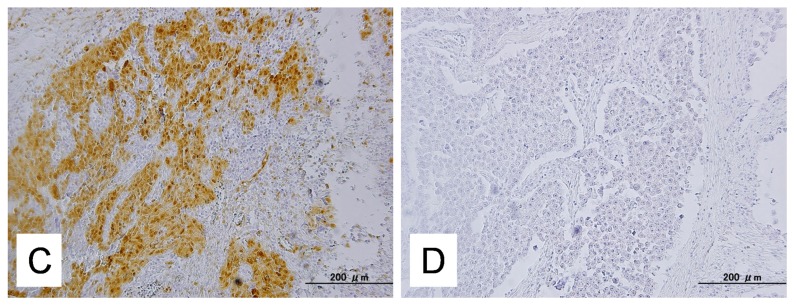
Dual-color fluorescence *in situ* hybridization (FISH) validates amplification of the *KRAS* or *MAPK1* gene in type II ovarian carcinoma. (**A**) FISH analysis of *KRAS* showed a homogeneously stained region in a tumor with gene amplification. White arrows indicated amplification of *KRAS* gene; (**B**) FISH analysis of *MAPK1* showed a homogeneously stained region in a tumor with gene amplification. White arrows indicated amplification of *MAPK1* gene; (**C**) Intense immunoreactivity toward p-ERK was present in both the nuclei and cytoplasm of carcinoma cells; (**D**) This sample shows negative case of staining for p-ERK.

*KRAS* amplification was identified in 9 (13.2%) of 68 type II ovarian carcinoma tissue samples, while *MAPK1* amplification was identified in 5 (7.4%) of 68 type II ovarian carcinoma tissue samples (Figure 1). Interestingly, co-amplification of *KRAS* and *MAPK1* almost absent in the tested type II ovarian carcinomas, except one case. The immunoreactivity of active p-ERK1/2 was detected in both the nucleus and the cytoplasm of the tumor cells (Figure 1). This is consistent with an earlier report [7,8]. Active p-ERK1/2 was identified in 26 (38.2%) of 68 high-grade serous ovarian carcinomas. Amplification of *KRAS* or *MAPK1* did not correlate with p-ERK immunoreactivity (Table 1).

### 2.2. Relationship between *KRAS* or *MAPK1* Amplification or p-ERK1/2 Expression and Clinicopathological Factors

Patients were stratified into two groups depending on the amplification status of *KRAS* or *MAPK1*. Patients were also stratified into two groups depending on the p-ERK1/2 expression. The relationships between *KRAS* or *MAPK1* amplification or p-ERK1/2 expression and clinicopathological factors are shown in Table 2. There was no significant correlation between *KRAS* or *MAPK1* amplification or p-ERK1/2 expression and the patient age, FIGO stage, or status of residual disease (Table 2).

### 2.3. Effects of *KRAS* or *MAPK1* Amplification and p-ERK1/2 Status on the Prognosis of Type II Ovarian Carcinomas

**Figure 2:**
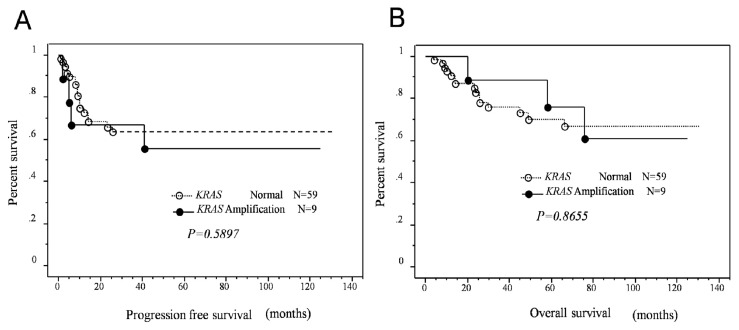

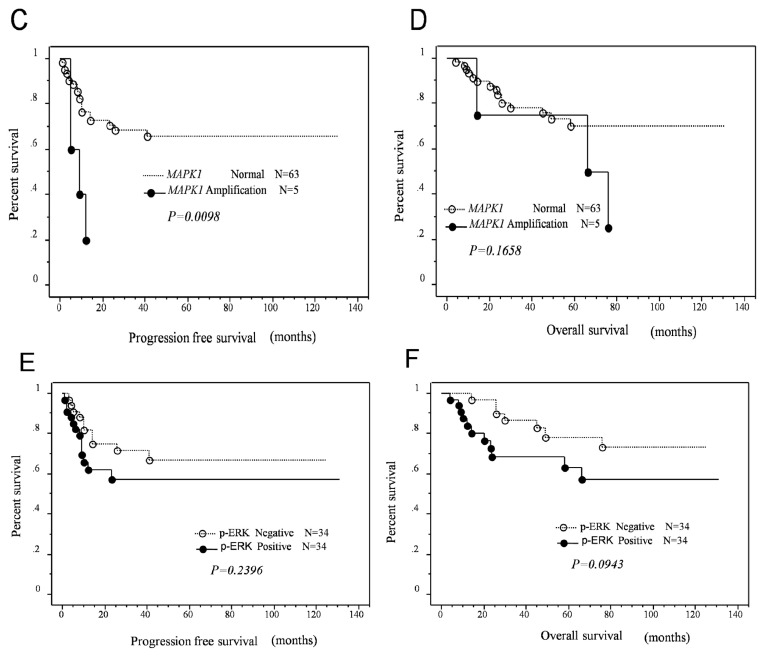
Relationship between *KRAS*/*MAPK1* amplification or p-ERK expression status and progression-free/overall survival in patients who received primary cytoreductive surgery followed by standard platinum/taxane chemotherapy. (**A**,**B**) Kaplan-Meier survival analysis showed that *KRAS* amplification (solid line, *n* = 9) was not associated with a shorter progression-free/overall survival than absence of *KRAS* amplification (dashed line, *n* = 59; *p = 0.5897*, *p = 0.8655*, respectively, Log-rank test); (**C**) Kaplan-Meier survival analysis showed that *MAPK1* amplification (solid line, *n* = 5) was associated with a shorter progression-free survival than absence of *MAPK1* amplification (dashed line, *n* = 63; *p* = 0.0098, Log-rank test); (**D**) MAPK1 amplification (solid line, *n* = 5) tended to be correlated with a shorter overall survival than absence of *MAPK1* amplification, but was not statistically significant (dashed line, *n* = 63; *p* = 0.1658, Log-rank test); (**E**,**F**) Positive p-ERK expression tended to be correlated with a shorter progression-free/overall survival than absence of *MAPK1* amplification, but was not statistically significant (dashed line, *n* = 63; *p* = 0.2396, *p* = 0.0943, respectively, Log-rank test).

Next, we examined the prognostic effects of *KRAS* or *MAPK1* amplification and p-ERK1/2 expression. Kaplan-Meier estimates of progression-free and overall survival are plotted in Figure 2. There was no significant relationship between *KRAS* amplification and overall or progression-free survival in patients with type II ovarian carcinoma (*p* = 0.2460, *p* = 0.9339, respectively; Figure 2). Patients with *MAPK1* amplification tended to have a poor overall survival; however, the difference was not statistically significant. In contrast, patients with *MAPK1* amplifications had significantly poorer progression-free survival than patients without *MAPK1* amplification.

Univariate analysis demonstrated that FIGO stage III–IV (*p* = 0.002; log-rank test), patient age (>60 years; *p* = 0.049; log-rank test), residual tumor ≥1 cm (*p* < 0.0001; log-rank test), and *MAPK1* amplification (*p* = 0.0098; log-rank test) correlated with shorter progression-free survival. When data were stratified for multivariate analysis, *MAPK1* amplification, patient age, and residual tumor ≥1 cm remained significantly associated with shorter progression-free survival (*p* = 0.029, *p* = 0.044, *p* = 0.003, respectively; Table 3). Patients with positive p-ERK expression in type II ovarian carcinomas tended to have a poor progression-free/overall survival; however, the difference was not statistically significant (*p* = 0.127, *p* = 0.087, respectively; Figure 2).

### 2.4. Identification of *KRAS* Mutations

Out of the 68 samples examined, 51 were available for mutation analysis. Somatic mutations in *KRAS* were identified in 3 (5.8%) of 51 type II ovarian carcinomas. All *KRAS* mutations were located at codon 12. No cases with concomitant *KRAS* or *MAPK1* amplification and *KRAS* mutation were identified. We previously reported the KRAS mutation profiles of type II ovarian carcinoma cell lines [8]; these data were used for subsequent *in vitro* analysis.

### 2.5. Effects of ERK1/2 Inactivation on High-Grade Serous Ovarian Carcinoma *in Vitro*

**Figure 3:**
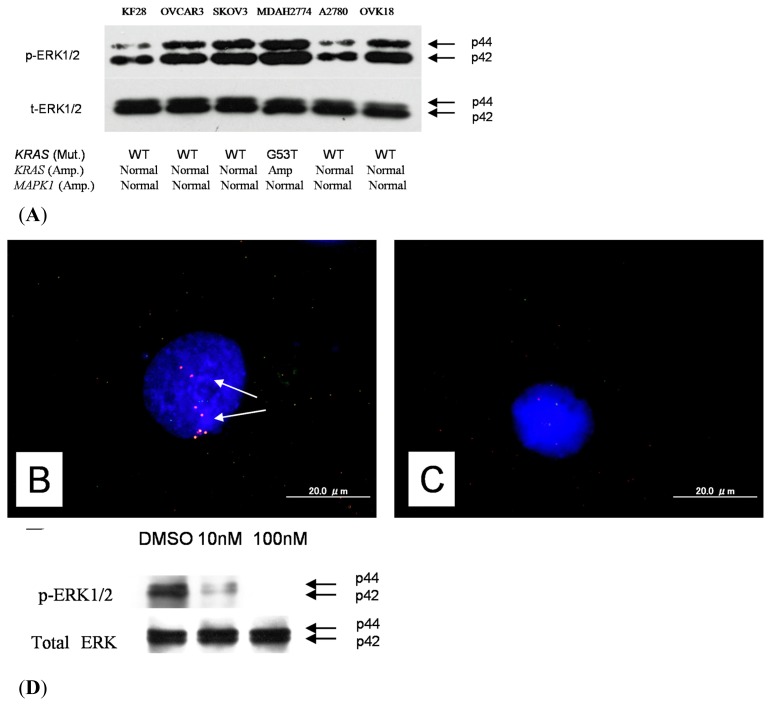

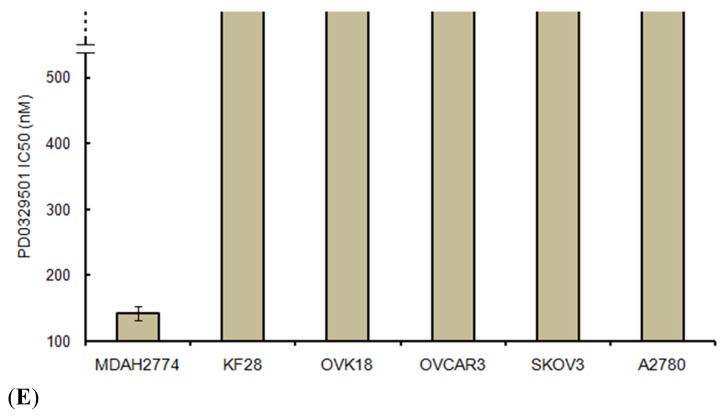
Western blot analysis. (**A**) Western blot analysis showed a higher level of p-ERK protein expression in MDAH2774 cells than in other cell lines; (**B**) Dual-color fluorescence *in situ* hybridization (FISH) validated amplification of the *KRAS* gene in ovarian cancer cell line (MDAH2774) White arrows indicate amplification of *KRAS* gene; (**C**) OVCAR3 cells contained signals for both *KRAS* and reference probes with an approximate 1:1 ratio; (**D**) Western blot analysis showed a significant reduction in p-ERK protein in PD0328901-treated cells compared with control DMSO-treated cells; (**E**) IC_50_ values for the selective MEK1/2 inhibitor PD0325901 for 6 type II ovarian carcinoma cell lines. MDAH2774 cells with *KRAS* amplification were more sensitive to growth inhibition by the selective MEK1/2 inhibitor PD0325901 than cells without *KRAS* amplification. The mean and SD were obtained from 3 experiments.

A panel of type II ovarian carcinoma cell lines was analyzed for *KRAS or MAPK1* amplification; as shown in Figure 3, 1 type II ovarian carcinoma cell line harbored *KRAS* amplification. Interestingly, the MDAH2774 cell line had both *KRAS* amplification and *KRAS* mutations. Amplification status was correlated with growth inhibition induced by the selective MEK inhibitor PD0325901, which prevented activation of the downstream target, ERK1/2. Western blot analysis showed a dose-dependent effect on the expression of p-ERK1/2 in MDAH2774 cells, and p-ERK1/2 was not detectable 1 h after treating the cells with PD0325901 at a concentration of 10 nM (Figure 3). As shown in Figure 3, MDAH2774 cells harboring either *KRAS* amplification or *KRAS* mutations showed a markedly lower IC_50_ for PD0325901.

PD0325901 had no significant effects on the growth of normal cells, including OSE cells (data not shown). It is likely that *KRAS*/*MAPK1* amplification or *KRAS* mutations are not the only determinant for the activation of ERK1/2. Therefore, we analyzed p-ERK1/2 expression in each of the cell lines listed in Figure 3. Only 1 cell line, MDAH2774, strongly expressed p-ERK1/2. SKOV3 and OVCAR3 showed moderate expression of ERK1/2. The other cell lines (KF28, A2780, and OVCA18) showed weak expression of ERK1/2. These results suggested that activation of ERK1/2 may not depend on *KRAS*/*MAPK1* amplification or *KRAS* mutations in type II ovarian carcinoma cell lines.

## 3. Discussion

Type II ovarian carcinomas are the most common and fatal form of ovarian cancer [9]. While most tumors are highly sensitive to cytoreductive surgery and platinum-and taxane-based chemotherapy, the majority of patients experience recurrence of treatment-resistant tumors. Therefore, a new potential molecular targeted therapy for type II ovarian carcinomas needs to be developed. We reported earlier that *KRAS* or *BRAF* mutations were quite common in low-grade serous ovarian carcinomas with prototypic histology of type I ovarian carcinoma but rare in high-grade serous ovarian carcinomas [8,10]. Our present results showing low frequencies of *KRAS* mutations in type II ovarian carcinoma are consistent with our earlier reports [8,10].

Based on our combined FISH and immunohistochemical analysis, we found that p-ERK was not consistently expressed in all *KRAS* or *MAPK1* gene amplification tumors. Why does this discrepancy happen? This may be due to expression of the mitogen-activated protein kinase (MAPK) phosphatase (MKP)-1 in type II ovarian carcinomas. MKP-1 is overexpressed in several types of cancers [11,12]. MKP-1 is the prototypic member of a family of dual-specificity phosphatases that dephosphorylate tyrosine and threonine residues on target proteins. This phosphatase is best known for its specificity toward p44/42 MAPK [13]. Further examination is needed to clarify the relationship *KRAS/MAPK1* amplification and MKP-1 expression. Furthermore, many tumors exhibited positive p-ERK signals in the absence of *KRAS* or *MAPK1* amplification. Therefore, gene amplification is probably just one of multiple mechanisms for p-ERK expression. Additionally, the low frequency of *KRAS* mutation or *BRAF* mutations in type II ovarian carcinomas in the current and previous studies suggest that p-ERK expression may be affected by the other receptor tyrosine kinase regulation mechanisms (*i.e.*, EGFR, Her2, *etc.*) [8,14,15]. This is in consistent with a recent report showing that this pathway is frequently activated independent of the status of *KRAS* and *BRAF* in endometrioid-type endometrial cancer [11]. The RAS–RAF–MEK–ERK pathway may be affected by multiple pathways in type II ovarian carcinogenesis and endometrial carcinogenesis. For example, alternative pathways for ERK activation, such as crosstalk with the PI3K pathway, exist in high-grade serous ovarian carcinomas. Indeed, enhanced PI3K signaling due to amplification occurs in 10%–18% of high-grade serous ovarian carcinomas, which is the dominant histology of type II ovarian carcinoma [6,10,16].

Biomarkers that can predict clinical prognosis, including treatment response and overall survival, can have a substantial clinical impact on the management of patients with ovarian carcinoma [17]. To further explore the clinical relevance of *KRAS* or *MAPK1* alterations in type II ovarian carcinomas, we identified a correlation between *KRAS* or *MAPK1* amplification or p-ERK protein expression and length of progression-free/overall survival in patients with type II ovarian carcinomas. Interestingly, a strong correlation between poor progression-free prognosis and *MAPK1* amplification was identified in patients who received taxane- and platinum-based chemotherapy. In contrast to *MAPK1* amplification, *KRAS* amplification did not affect patient survival. However, p-ERK protein overexpression was associated with an insignificant trend toward poor progression-free/overall survival. The mechanism underlying the association between *MAPK1* amplification and shorter progression-free survival is not known; however, because mortality of patients with type II ovarian carcinomas is directly related to recurrence of disease after chemotherapy, it is conceivable that *MAPK1* amplification may confer resistance to chemotherapy and/or enhance cell proliferation in recurrent chemoresistant tumors. Current study has several limitations. First, differences in histological heterogeneity in type II ovarian carcinoma, including high-grade serous, high-grade endometriod, and carcinosarcoma used to assess amplification *KRAS/MAPK1* may produce significant variability among results. Second, the sample sizes of this study are relatively small. Larger sample studies are required to definitively establish the percentage of *KRAS/MAPK1* amplification on type II ovarian carcinomas.

In the current study, type II ovarian carcinomas with both *KRAS* mutations and *KRAS* amplification were highly sensitive to growth inhibition by the selective MEK inhibitor, PD0325901. This observation suggested that type II ovarian carcinoma with concomitant amplification and mutation of *KRAS* was more highly dependent on the activation of the RAS/RAF/MEK/ERK pathway for cell proliferation and survival than those without such amplification or mutation. Thus, inactivation of ERK1/2 resulted in marked growth inhibition in ovarian carcinomas with amplification/mutations in *KRAS* in comparison with only a slight effect on wild-type tumors. The above observations lend strong support to the view of ‘oncogene addiction’ [18] by which the activating amplifications in the kinase pathway confer susceptibility of the tumors to an inhibitor [8,19,20]. Recently, Wagner *et al.* reported that concomitant *KRAS* amplification and mutation enhances the aggressiveness of non-small cell lung carcinoma [21]. In contrast, in the current study no cases with concomitant *KRAS* or *MAPK1* amplification and *KRAS* mutation were identified. This discrepancy may be due to differences in organ-specific oncogenic pathways and mechanisms of carcinogenesis. The potential for organ-specific differences in *KRAS* amplification and *KRAS* mutations in malignant tissues is the subject of ongoing investigation. Although our *in vitro* results showed that concomitant *KRAS* amplification and mutation was more highly dependent on the activation of the RAS/RAF/MEK/ERK pathway for cell proliferation than those without such amplification or mutation. Considering the above findings, concomitant *KRAS* amplification/mutation and function in neoplastic tissues may be dependent on cellular context. In order to fully understand the role of the complex concomitant amplification and mutation of *KRAS* in type II ovarian carcinoma physiology, larger studies including functional analyses are required.

## 4. Experimental Section

### 4.1. Tissue Samples

Formalin-fixed, paraffin-embedded tissue samples from 68 type II ovarian carcinomas, including 43 high-grade serous, 16 high-grade endometrioid, and 5 carcinosarcoma tumors, were used in this study. Samples were obtained from the Department of Obstetrics and Gynecology at the Shimane University Hospital. Diagnosis was based on conventional morphological examination of sections stained with hematoxylin and eosin (H & E), and tumors were classified according to the WHO classification. Tumor staging was performed according to the International Federation of Gynecology and Obstetrics (FIGO) classification. All patients were primarily treated with cytoreductive surgery and adjuvant platinum and taxane chemotherapy (CBDCA AUC5 with paclitaxel 175 mg/m^2^ or docetaxel 70 mg/m^2^). All patients received 6–12 courses of this combination regimen. The acquisition of tumor tissues was approved by the Shimane University Institutional Review Board. Paraffin tissue blocks were organized into tissue microarrays, each made by removing tumor cores measuring 3 mm in diameter from the block. Selection of the area to core was made by a gynecologic oncologist (K.N.) and pathology technician (K.I.) and was based on review of the H & E slides.

### 4.2. Fluorescence *in Situ* Hybridization

BAC clones (CTD2174F1 and CTD-2536C1) containing the genomic sequences of the 12p12.1 chromosome at the *KRAS* locus were purchased from Bacpac Resources (Children’s Hospital, Oakland, CA, USA) and Invitrogen (Carlsbad, CA, USA). Bac clones located at Chr12q23.3 (RP11-482D24) were used as reference probes. BAC clones (RP11-647D11 and RP11-1109D18) containing the genomic sequences of the 22q11.21–22q11.22 chromosome at the *MAPK1* locus were purchased from Bacpac Resources and Invitrogen. Bac clones located at Chr22q13.32-13.33 (RP11-29C18) were used as reference probes.

CTD2174F1, CTD-2536C1, RP11-647D11, and RP11-1109D18 were labeled by nick translation with biotin-dUTP; RP11-482D24 and RP11-29C18 were labeled similarly with digoxigenin-dUTP. To detect biotin-labeled and digoxigenin-labeled signals, slides were first incubated with FITC-avidin (Vector Laboratories, Burlingame, CA, USA) and anti-digoxigenin mouse antibodies (Roche Molecular Biochemicals, Mannheim, Germany). Slides were subsequently incubated with biotinylated anti-avidin antibodies (Vector Laboratories, Burlingame, CA, USA) and tetramethylrhodamine B isothiocyanate (TRITC)-conjugated rabbit anti-mouse antibodies (Sigma, St. Louis, MO, USA). The final incubation was with FITC-avidin and TRITC-conjugated goat anti-rabbit antibodies (Sigma, St. Louis, MO, USA). Slides were counterstained with 4′,6′-diamidino-2-phenylindole (DAPI, Sigma, St. Louis, MO, USA).

Fluorescence *in situ* hybridization (FISH) signals were evaluated with an Olympus fluorescence microscope BX41 (Tokyo, Japan) by two individuals who were blinded to the treatment history of each patient. Separate narrow band pass filters were used for detection of TRITC, FITC, and DAPI signals. Using a 60× objective lens, approximately 100 tumor cells were examined for each specimen, and the number of fluorescent signals within tumor cells from *KRAS or MAPK1* gene BAC probes and chromosome 12q23.3 or 22q13.32-13.33 reference BAC probe was recorded. Amplification of *KRAS* or *MAPK1* was defined as a ratio of *KRAS or MAPK1* BAC probe signals to chromosome 12 or chromosome 22 centromeric reference BAC probe signals of 2:1 or more.

### 4.3. Mutational Analysis of *KRAS*

Genomic DNA was purified from all cell lines and formalin-fixed, paraffin-embedded tissues using a Qiaquick polymerase chain reaction (PCR) purification kit (Qiagen, Valencia, CA, USA). PCR was then carried out followed by nucleotide sequencing using the iCycler (Bio-Rad, Hercules, CA, USA). Exon 1 of *KRAS* was sequenced, as these mutational hot spots together harbor nearly all published mutations [22,23]. The primers for PCR and sequencing were manufactured by GeneLink (Hawthorne, NY, USA), and their sequences were described in an earlier report [9]. The sequences were analyzed using the Lasergene program, DNASTAR (Madison, WI, USA).

### 4.4. Immunohistochemistry

Expression of active phosphorylated ERK1/2 (p-ERK1/2) was assessed by immunohistochemistry and western blot analysis. The antibody used in this study was a rabbit polyclonal antibody that reacted with phosphorylated but not unphosphorylated ERK1/2 (Cell Signaling Technology, Danvers, MA, USA). Immunohistochemistry was carried out on tissue microarrays at a dilution of 1:1000 followed by detection with the En Vision+ System using the peroxidase method (DAKO, Carpinteria, CA, USA). The percentage of positive cells was estimated by randomly counting ~500 tumor cells from 3 different high-power fields (40×) within one specimen. A positive reaction was defined as a discrete localization of the brown chromagen in the nucleus or cytoplasm. Cases in which more than 5% of the tumor cells showed detectable immunoreactivity were scored as positive.

### 4.5. Western Blot Analysis

Cell lysates were prepared by dissolving cell pellets in Laemmli sample buffer (Bio-Rad) supplemented with 5% β-mercaptoethanol (Sigma). Western blot analysis was performed on type II ovarian carcinoma cell lines, including OVCAR3, SKOV3, A2780, MDAH2774, KF28, and OVK18. Similar amounts of total protein from each lysate were loaded and separated on 10% Tris-Glycine-SDS polyacrylamide gels (Novex, San Diego, CA, USA) and electroblotted to Millipore Immobilon-P polyvinylidene difluoride membranes. The membranes were probed with p-ERK1/2 antibodies (pTEpY, 1:1000; Santa Cruz Biotechnology, CA, USA) followed by peroxidase-conjugated anti-mouse immunoglobulin (1:10,000). The same membrane was probed with an antibody that reacted with total ERK1/2 (1:5000; Santa Cruz Biotechnology, CA, USA) as a loading control. Western blots were developed by chemiluminescence (Pierce, Rockford, IL, USA).

### 4.6. Cell Growth Assays

For cell growth assays, cells were plated at the same density (5 × 10^3^ cells per well) in 96-well plates. A methyl thiazoyl tetrazorium (MTT) cell growth assay was performed [24] 96 h after treating the cells with PD0325901 (Selleck Chemicals, Houston, TX, USA) at 1–200 nM or with dimethyl sulfoxide (DMSO) as a control. The IC_50_ was determined on the basis of dose-response curves by a cytotoxicity assay. The data were expressed as the mean ± SD of triplicates.

### 4.7. Statistical Methods for Clinical Correlations

Overall survival was calculated from date of diagnosis to date of death or last follow-up. Progression-free survival was defined as the time from the first day of surgery or chemotherapy until the first of either death from any cause or disease progression (based on an increase in the CA 125 levels and/or on the findings of imaging studies). Survival data were plotted as Kaplan-Meier curves, and statistical significance was determined by the Log-rank test. Multivariate prognostic analysis was performed using a Cox proportional hazards model. Data were censored when patients were lost to follow-up. The chi-square test or Fischer’s exact test was used for comparisons of categorical data.

## 5. Conclusions

In summary, we demonstrated that phenotypic changes in type II ovarian carcinomas in response to ERK1/2 inactivation depended on the amplification status of *KRAS*/*MAPK1* or the mutation status of *KRAS*. The findings in this study provide new insights into the biological roles of the RAS/RAF/MEK/ERK signaling pathway in type II ovarian carcinomas. In addition, our observations have important therapeutic implications in the treatment of type II ovarian carcinoma in patients harboring *KRAS* or *MAPK1* amplifications. Ovarian carcinomas with *KRAS* or *MAPK1* amplifications are clinically most frequent in type II carcinomas that exhibit aggressive behavior [5,6]. Therefore, detection of *KRAS* and *MAPK1* amplifications in type II ovarian carcinomas may identify patients who will benefit from therapy with the selective MEK inhibitor PD0325901.

## Figures and Tables

**Table 1:** Association between *KRAS/MAPK1* amplification and p-ERK expression.

	p-ERK negative	p-ERK positive
*KRAS/MAPK1* normal	29	26
*KRAS/MAPK1* amplification	5	8
		*p* = 0.5387

**Table 2:** Association between *KRAS* or *MAPK1* gene amplification or p-ERK expression and clinicopathological factors in patients with type II ovarian carcinomas.

Factors	Patients	*KRAS* FISH		*p-*value	*MAPK1* FISH		*p-*value	p-ERK imunostaining		*p-*value

	(*n*)	Normal	Amplification		Normal	Amplification		Negative	Positive	
FIGO stage										

I, II	31	26	5	0.5192	30	1	0.2327	14	17	0.4651
III, IV	37	33	4		33	4		20	17	

Age (years)										

<60	32	26	6	0.2058	28	4	0.1252	15	17	0.627
≥60	36	33	3		35	1		19	17	

Residual tumor										

<1 cm	39	32	7	0.1835	38	1	0.0793	19	20	0.8063
≥1 cm	29	27	2		25	4		15	14	

**Table 3:** Univariate and multivariate analysis of progression-free prognostic factors in patients with type II ovarian carcinomas.

Factors	Patients	Univariate			Multivariate		
		
		hazard ratio	95% CI	*p-*value	hazard ratio	95% CI	*p-*value
FIGO stage							

I, II	31	7.1	2.1–24.1	0.0015	1.3	0.3–5.8	0.7511
III, IV	37						

Age (years)							

<60	32	2.5	1.0–6.0	0.0499	2.8	1.0–7.7	0.0438
≥60	36						

Residual tumor							

<1 cm	39	14.2	4.1–48.2	<0.0001	10.2	2.2–46.7	0.0028
≥1 cm	29						

*MAPK1* FISH							

Amplification	5	4.4	1.4–1.3	0.0098	4.2	1.2–15.0	0.0285
Normal	63						
